# Endothelial deletion of TBK1 contributes to BRB dysfunction via CXCR4 phosphorylation suppression

**DOI:** 10.1038/s41420-022-01222-y

**Published:** 2022-10-28

**Authors:** Bowen Zhao, Yueqi Ni, Hong Zhang, Yin Zhao, Lu Li

**Affiliations:** 1 Department of Ophthalmology, Tongji Hospital, Tongji Medical College, Huazhong University of Science and Technology, Wuhan, 430030 People’s Republic of China; 2 Department of Ophthalmology, Affiliated Wuxi Clinical College of Nantong University, Wuxi, 214000 People’s Republic of China; 3 Department of Ophthalmology, The Affiliated Wuxi No. 2 People’s Hospital of Nanjing Medical University, Wuxi, 214000 People’s Republic of China

**Keywords:** Retinal diseases, Phosphorylation

## Abstract

Blood-retinal barrier (BRB) dysfunction has been recognized as an early pathological feature in common eye diseases that cause blindness. The breakdown of endothelial cell-to-cell junctions is the main reason for BRB dysfunction, yet our understanding of junctional modulation remains limited. Here, we demonstrated that endothelial-specific deletion of TBK1 (*Tbk1*^*ΔEC*^) disrupted retinal vascular development, and induced vascular leakage. LC-MS/MS proteomic analysis was used to identify candidate substrates of TBK1. We found that TBK1 interacted with CXCR4, and the phosphorylation level of CXCR4-Serine 355 (Ser355) was decreased in *Tbk1*^*ΔEC*^ retina samples. Furthermore, TBK1-mediated phosphorylation of CXCR4 at Ser355 played an indispensable role in maintaining endothelial junctions. Interestingly, we also detected an increased expression of TBK1 in diabetic retinopathy samples, which suggested an association between TBK1 and the disease. Taken together, these results provided insight into the mechanisms involved in the regulation of endothelial cell-to-cell junctions via TBK1-dependent CXCR4 phosphorylation.

## Introduction

Age-related macular degeneration (AMD) and diabetic retinopathy (DR) are the leading causes of vision loss in elderly and working-age individuals, respectively. The breakdown of BRB serves as one of the direct pathogenetic mechanisms in AMD and DR. The BRB restricts the entry of macromolecules and other potentially harmful agents into the retina, and BRB integrity is crucial for the homeostasis of the retina [1]. The endothelium is a core component of the BRB, which exhibits highly organized intercellular junctions. Great efforts have been made to understand the regulation of BRB and endothelial junctions under physiological or/and pathological conditions.

TANK-binding kinase 1 (TBK1) belongs to the non-canonical IκB kinases, and is also known as an NF-κB activating kinase [2]. TBK1 can be activated by pro-inflammatory cytokines and viral infections. TBK1 knockdown resulted in a dramatic reduction in the activation of the primary antiviral response [3]. Furthermore, the involvement of TBK1 expression in vessel formation has been uncovered. A previous study found that TBK1 correlated with endothelial proliferation [4]. The expression of TBK1 was upregulated in a laser-induced mouse choroidal neovascularization (CNV) model, whereas anti-TBK1 antibody treatment could ameliorate CNV formation [5]. However, the role of TBK1 in BRB, especially in endothelial junctional integrity, is still unclear.

The Chemokine receptor, C-X-C chemokine receptor 4 (CXCR4), also known as CD184, is a G protein-coupled receptor. CXCR4 is expressed in various cell types, including endothelial cells [6], neurons [7], and glial cells [8]. CXCR4 can be activated by stromal cell-derived factor-1 (also known as CXCL12) and macrophage migration inhibitory factors. CXCR4 activation and signal transduction is also mediated through phosphorylation by specific protein kinases. Previous studies have identified that CXCR4 could be phosphorylated at Ser324/5 and Ser346/7 by protein kinase C [9, 10].

In this study, we reported that endothelial-specific deletion of TBK1 destroyed vascular stabilization during development and increased vascular permeability. Based on phosphoproteomic dataset analysis, we have uncovered CXCR4 as a novel candidate substrate of TBK1. Deletion of TBK1 significantly decreased the phosphorylation level of CXCR4 Ser355. TBK1 knockdown disrupted endothelial tight junctions and adherent junctions, which were maintained by phosphorylated CXCR4 Ser355. In addition, we have found an increased expression of TBK1 in DR samples and high glucose-stimulated endothelial cells. In general, our results suggest that targeting TBK1-CXCR4 may be a new strategy for protecting BRB under pathological conditions.

## Results

### Endothelial TBK1 deprivation affected retinal vascular development

**Fig. 1:**
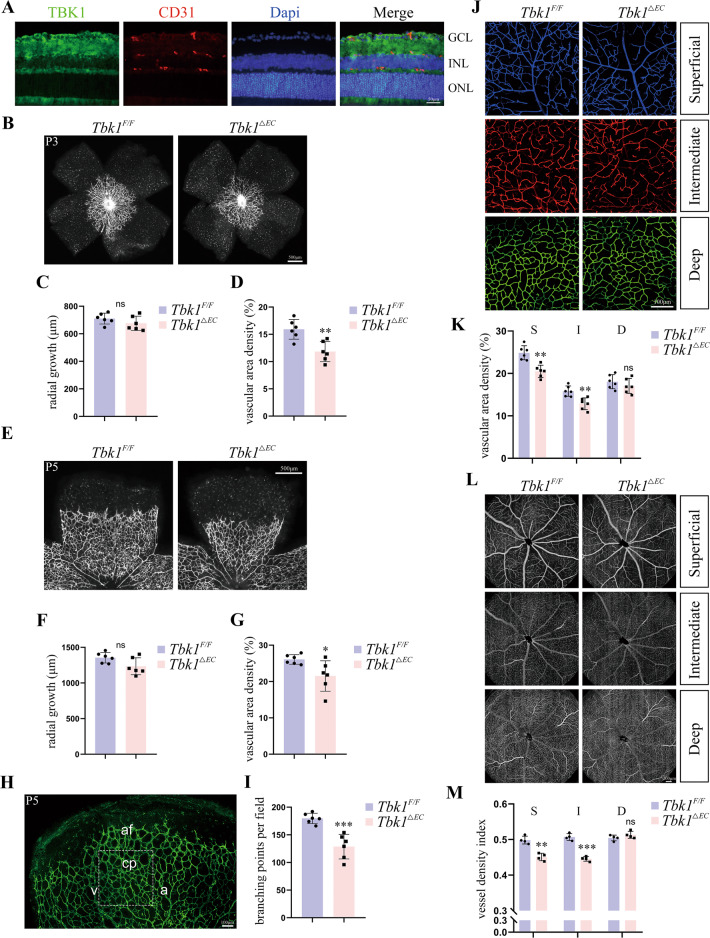
The effect of endothelial TBK1 deletion on retinal vascular development. **A** Immunofluorescence images of retinal cross-section stained with TBK1 and CD31. **B** Whole-mount IB4 labeling of retinal vasculature of P3 *Tbk1*^*F/F*^ and *Tbk1*^*ΔEC*^ littermates. **C**, **D** Quantification of vascular radial growth (**C**) and vascular density (**D**) in **B**. The data were means ± SD of six retinas/group and assessed by student’s *t*-test. **E** Whole-mount IB4 labeling of retinal vasculature of P5 *Tbk1*^*F/F*^ and *Tbk1*^*ΔEC*^ littermates. **F** Quantification of vascular radial growth in **E**. The data were means ± SD of six retinas/group and assessed by student’s *t*-test. **G** Quantification of vascular density in **E**. The data were means ± SD of six retinas/group and assessed by Welch’s *t*-test. **H** Representation of the region (dashed-line square) where branching points were analyzed. Indicating a capillary plexus close to the edge of the angiogenic front between an arteriole and a venule. af angiogenic front, cp capillary plexus, a arteriole, v venule. **I** Quantification of branching points in capillary plexus of P5 *Tbk1*^*F/F*^ and *Tbk1*^*ΔEC*^ mice. The data were means ± SD of six retinas/group and assessed by student’s *t*-test. **J** Representative whole-mount IB4 labeling of retinal vasculature of P18 *Tbk1*^*F/F*^ and *Tbk1*^*ΔEC*^ littermates. Pseudo color for distinguishing superficial, intermediate, and deep plexuses. **K** Quantification of vascular density in **J**. The data were means ± SD of six retinas/group and assessed by multiple *t*-test. **L** OCTA images of retinal vascular plexuses in adult *Tbk1*^*F/F*^ and *Tbk1*^*ΔEC*^ mice. **M** Analysis of vessel density index in vascular plexuses in **L**. The data were means ± SD of four retinas/group and assessed by multiple *t*-test. *P < 0.05, **P < 0.01, ***P < 0.001.

*Tbk1*^*flox/flox*^ (*Tbk1*^*F/F*^) mice were crossed with *Tie2-Cre* mice to establish a vascular endothelial cell-specific TBK1 knockout model (*Tbk1*^*ΔEC*^). Endothelial TBK1 deletion was proved by retinal cross-sectional immunofluorescence shown in Fig. 1A. Firstly, we evaluated the effect of endothelial TBK1 deletion on vessel sprouting in newborn mice. No significant difference was detected in vascular radial growth between *Tbk1*^*F/F*^ and *Tbk1*^*ΔEC*^ infants in postnatal day (P) 3 (Fig. 1B, C) and P5 (Fig. 1E, F). Otherwise, endothelial TBK1 deprivation induced decreased vascular density in both P3 (Fig. 1B, D) and P5 retinas (Fig. 1E, G). Moreover, fewer branching points were detected in the retinal capillary plexus in P5 *Tbk1*^*ΔEC*^ infants compared to control littermates (Fig. 1H, I). Then, we focused on the vascular density in P18 retinas, where all the vascular plexuses were completely developed (Fig. 1J). *Tbk1*^*ΔEC*^ group showed decreased vascular density in both superficial and intermediate plexuses, but not in the deep plexus (Fig. 1K). To evaluate the vascular density in adulthood in vivo, optical coherence tomography angiography (OCTA) scanning was performed on *Tbk1*^*F/F*^ and *Tbk1*^*ΔEC*^ retinas (Fig. 1L). We found that endothelial TBK1 deprivation induced decreased vessel density in superficial and intermediate plexuses (Fig. 1M), in coincidence with the results at the time point P18. Collectively, compromised vascular density was detected in *Tbk1*^*ΔEC*^ mice during retinal vascular development. As previously reported, TBK1 promoted endothelial proliferation and was indispensable for vascularization [4], which could provide an explanation for the phenotypes detected in *Tbk1*^*ΔEC*^ mice in our study.

### Endothelial TBK1 deprivation destroyed vessel stability and barrier function

**Fig. 2:**
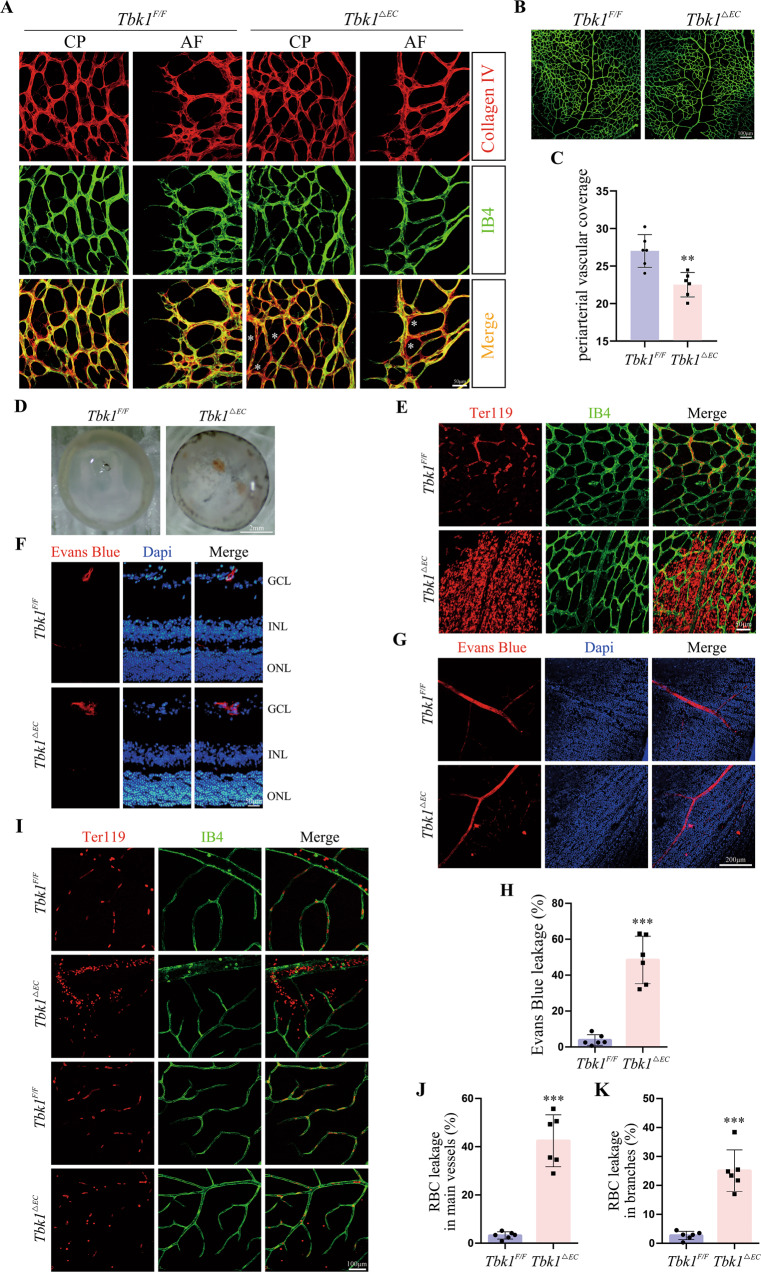
Endothelial TBK1 deprivation impairs BRB. **A** Representative retinal capillary plexus (CP) and angiogenic front (AF) of P5 *Tbk1*^*F/F*^ and *Tbk1*^*ΔEC*^ littermates. Vessel retraction was detected by increased collagen IV (+)/IB4(−) empty sleeves (asterisks) in *Tbk1*^*ΔEC*^ mutants. Three independent experiments were performed. **B** Whole-mount IB4 labeling of retinal periarterial vasculature of P7 *Tbk1*^*F/F*^ and *Tbk1*^*ΔEC*^ littermates. Note enlarged capillary-free area around *Tbk1*^*ΔEC*^ arteries. **C** Analysis of vascular coverage in **B**. The data were means ± SD of six retinas/group and assessed by student’s *t*-test. **D** Representative images of freshly dissected P7 retinas. Three independent experiments were performed. **E** Retinal whole-mount staining with TER119 and IB4 in P7 *Tbk1*^*F/F*^ and *Tbk1*^*ΔEC*^ mice. Red blood cells (RBCs) extravasation indicated BRB impairments in *Tbk1*^*ΔEC*^ mice. Three independent experiments were performed. **F** Representative cryosections of the retinas dissected 1 h after Evans Blue infusion (i.v.). The Dyes were restricted within vascular lumens in *Tbk1*^*F/F*^ adult mice, but permeating the surroundings in *Tbk1*^*ΔEC*^ adult mice. Three independent experiments were performed. **G** Whole-mount of the retinas dissected 1 day after Evans Blue infusion (i.v.) in adult *Tbk1*^*F/F*^ and *Tbk1*^*ΔEC*^ mice. **H** Quantification of vascular Evans Blue leakage in **G**. The data were means ± SD of six retinas/group and assessed by Welch’s *t*-test. **I** Retinal whole-mount staining with TER119 and IB4 in adult *Tbk1*^*F/F*^ and *Tbk1*^*ΔEC*^ mice. **J** Quantification of RBC leakage from retinal main vessels in **I**. The data were means ± SD of six retinas/group and assessed by Welch’s *t*-test. **K** Quantification of RBC leakage from retinal branches in **I**. The data were means ± SD of six retinas/group and assessed by Welch’s *t*-test. **P < 0.01, ***P < 0.001.

A vascular retraction was detected in *Tbk1*^*ΔEC*^ mutants, as shown by increased Collagen IV(+)/IB4(−) empty sleeves in P5 retinal capillary plexus and angiogenic front (Fig. 2A). Furthermore, we found excessive pruning of capillaries around developing arteries in *Tbk1*^*ΔEC*^ retinas (Fig. 2B), leading to compromised vascular coverage (Fig. 2C). Vessel instability resulted in vascular barrier dysfunction [11]. P7 *Tbk1*^*ΔEC*^ infants exhibited hemorrhage in the inner surface of the retina cup (Fig. 2D), which was validated by the extravasation of red blood cells (RBCs) from retinal vasculature (Fig. 2E). To evaluate BRB permeability in adult mice, Evans Blue was injected intravenously. Evans Blue leakage suggested BRB breakdown induced by endothelial TBK1 deletion (Fig. 2F–H). Consistently, RBCs extravasation was detected in retinal main vessels and branches in *Tbk1*^*ΔEC*^ mice (Fig. 2I–K).

### Experimental design and general features of quantitative phosphoproteomic dataset analysis

**Fig. 3:**
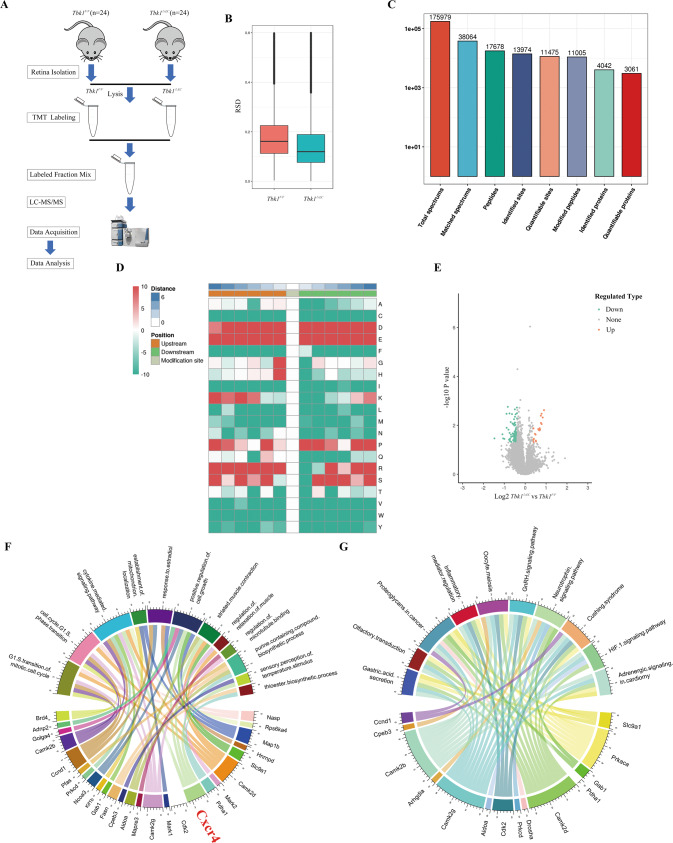
Overview of the phosphoproteomic analysis in *Tbk1*^*F/F*^ and *Tbk1*^*ΔEC*^ retinas. **A** Schematic outline of the workflow for quantitative phosphoproteomic analysis. **B** Relative standard deviation (RSD) for the *Tbk1*^*F/F*^ and *Tbk1*^*ΔEC*^ groups. **C** Numbers of the identified phosphopeptides and phosphorylated sites. **D** Motif analysis of the phosphopeptides with modified serine at position 0. **E** Volcano plot of phosphopeptide reporter ion intensity ratio (*Tbk1*^*ΔEC*^ vs *Tbk1*^*F/F*^) vs *p* value. Colored dots represented phosphopeptides with highly significant differential intensities. Red dots: upregulated; Green dots: downregulated. **F** GO Clustering analysis of proteins with different phosphorylation levels in the *Tbk1*^*ΔEC*^ group vs *Tbk1*^*F/F*^ group. **G** KEGG Clustering analysis of proteins with different phosphorylation levels in *Tbk1*^*ΔEC*^ group vs *Tbk1*^*F/F*^ group.

This study used TMT labeling and LC-MS/MS analysis to identify and quantify protein phosphorylation in retina samples of *Tbk1*^*F/F*^ and *Tbk1*^*ΔEC*^ P5 mice (Fig. 3A). To obtain sufficient protein for quantitative phosphoproteomic analysis, 24 mice per group were included, and therein 16 retinas from eight mice were assigned into a sample. Samples were labeled by TMT, and then were subjected to high-performance LC fractionation, affinity enrichment, and LC-MS/MS. There were no significant differences in relative standard deviation (RSD) between the two groups (Fig. 3B). A total of 11,005 modified peptides were identified and mapped to 3061 quantifiable proteins (Fig. 3C). Figure 3D showed motif analysis of the enrichment of phosphorylation centrally located at serine residues. Further statistical analysis indicated an increase in the phosphorylation level of 20 modification-sites, and a decrease in 104 phosphosites in *Tbk1*^*ΔEC*^ samples compared to the *Tbk1*^*F/F*^ group (Fig. 3E). As shown in Fig. 3F, G, clustering analyses linked the differentially expressed phosphoproteins to associated biological processes and pathways. Among the proteins, CXCR4 participates in cytokine-mediated signaling pathways and positive regulation of cell growth, which is shown to be related to endothelial/ vascular development [12].

### Clustering analyses of the differentially expressed phosphoproteins

**Fig. 4:**
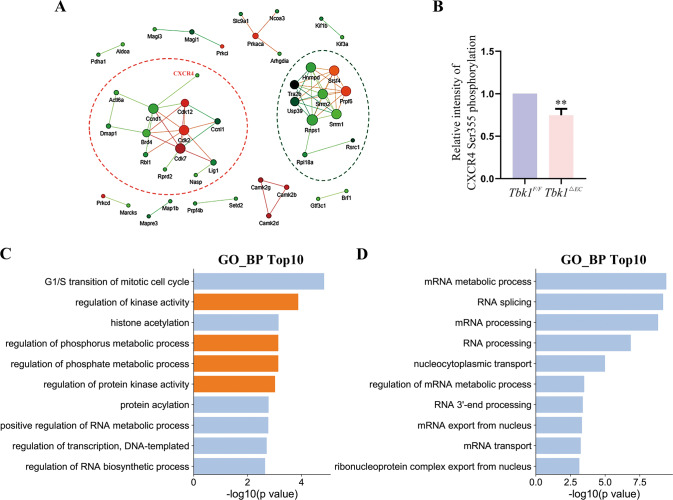
Bioinformatics analysis of the differentially expressed phosphoproteins. **A** Interactome of proteins with significantly different phosphorylation levels in the *Tbk1*^*ΔEC*^ group vs *Tbk1*^*F/F*^ group. Two interaction networks were identified: Network #1 with a red circle and Network #2 with a green circle. **B** Relative intensity of CXCR4 Ser355 phosphorylation in *Tbk1*^*F/F*^ and *Tbk1*^*ΔEC*^ retinas. The phosphorylation abundance was normalized with protein expression level. (*n* = 3; student’s *t*-test; **P < 0.01). **C** Histogram showing the top ten GO items of Network #1 in **A** from the STRING online database. Orange bars indicated the biological processes associated with CXCR4. **D** Histogram showing the top ten GO items of Network #2 in **A** from the STRING online database.

Within the differentially expressed phosphoproteins, two interaction networks were identified (Fig. 4A), and CXCR4 was a member of Network #1 (red circle). LC-MS quantitative analysis showed the phosphorylation level of CXCR4 Ser355 was notably reduced in *Tbk1*^*ΔEC*^ in comparison to *Tbk1*^*F/F*^ retinas (Fig. 4B). GO analysis was performed to investigate the biological processes of the TBK1 phosphosubstrates in both networks (Fig. 4C, D). CXCR4 is involved in the regulation of kinase activity, regulation of the phosphorus metabolic process, regulation of the phosphate metabolic process, and regulation of protein kinase activity (Fig. 4C).

### CXCR4 is phosphorylated by TBK1 at Ser355

**Fig. 5:**
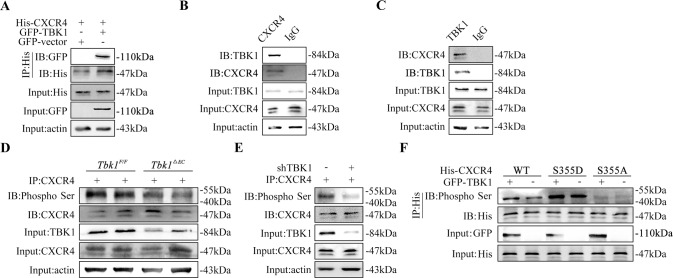
CXCR4 is phosphorylated by TBK1 at serine residues 355. **A** Coimmunoprecipitation (Co-IP) results showing the interaction of GFP-TBK1 with His-CXCR4 in HEK293T cells. Three independent experiments were performed. **B** Endogenous TBK1 was immunoprecipitated with CXCR4 in bEnd.3 cells. Three independent experiments were performed. **C** Endogenous CXCR4 was immunoprecipitated with TBK1 in bEnd.3 cells. Three independent experiments were performed. **D** CXCR4-serine phosphorylation level was decreased in *Tbk1*^*ΔEC*^ retinas, which was detected by immunoprecipitation (IP). Four independent experiments were performed. **E** IP assay showed a decrease in CXCR4-serine phosphorylation level in bEnd.3 cells transfected with shTBK1. Three independent experiments were performed. **F** The effect of CXCR4 Ser355 mutation on CXCR4-serine phosphorylation level in response to TBK1 overexpression in HEK293T cells. Three independent experiments were performed. actin: β-actin.

To validate whether CXCR4 can be phosphorylated by TBK1, HEK293T cells were transfected with GFP-TBK1 and His-CXCR4 plasmids. The interaction between TBK1 and CXCR4 was shown by coimmunoprecipitation (Co-IP) experiments (Fig. 5A). In endothelial cells, we found the endogenous interaction of TBK1 with CXCR4 (Fig. 5B, C). Then, retina tissue of *Tbk1*^*F/F*^ and *Tbk1*^*ΔEC*^ adults was isolated to evaluate CXCR4-serine phosphorylation level. Endothelial TBK1 deletion inhibited CXCR4 phosphorylation, as shown in Fig. 5D. Coincidentally, we detected decreased phosphorylation level of CXCR4-serine in endothelial cells transfected with shTBK1 (Fig. 5E). To examine whether TBK1 phosphorylated CXCR4 at Ser355, HEK293T cells were transfected with site-directed mutants of CXCR4 (phospho-mimetic S355D or phosphorylation-defective S355A) or wild-type CXCR4 (WT). As expected, TBK1 promoted the phosphorylation of CXCR4-WT, but had no effect on CXCR4 Ser355 mutants (Fig. 5F).

### Endothelial junctional disorganization arising from TBK1 knockdown was reversed by phosphorylated CXCR4 Ser355 complement

**Fig. 6:**
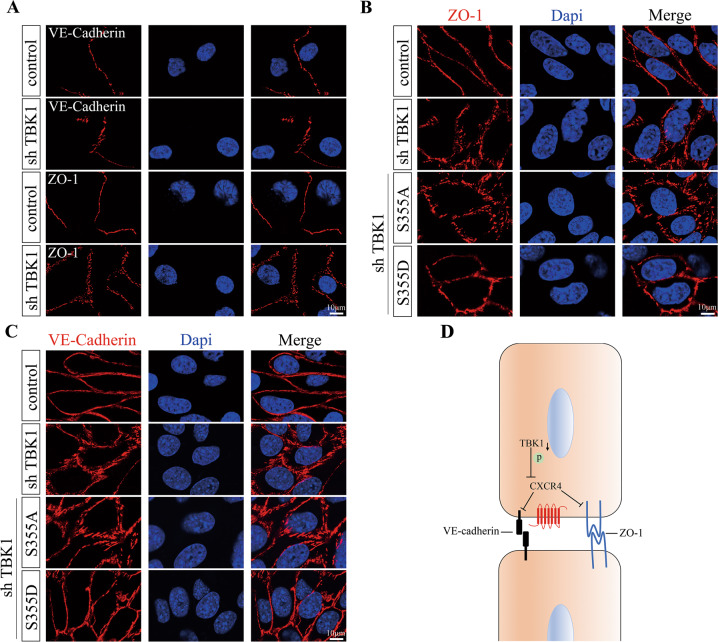
Endothelial TBK1 knockdown disrupted the organization of junctional proteins. **A** Representative images of VE-cadherin and ZO-1 organization in HRCECs transfected with shTBK1. Three independent experiments were performed. **B** Representative images of ZO-1 organization in bEnd.3 cells transfected with shTBK1 or together with CXCR4 mutant plasmids (S355A or S355D). Three independent experiments were performed. **C** Representative images of VE-cadherin organization in bEnd.3 cells transfected with shTBK1 or together with CXCR4 mutant plasmids (S355A or S355D). Three independent experiments were performed. **D** Illustration of the role of TBK1-CXCR4 in endothelial junctions.

Endothelial barrier dysfunction is characterized by the disorganization of junctional proteins [13]. In human retinal capillary endothelial cells (HRCECs), TBK1 inhibition induced loss of integrity and linearity in VE-cadherin and ZO-1 organization (Fig. 6A). The disruption of endothelial junctions was also detected in mouse brain microvascular endothelial cells (bEnd.3 cells) in response to TBK1-knockdown, as shown by the jagged and interrupted distribution of junctional proteins at EC-EC contacts (Fig. 6B, C). bEnd.3 cells complemented with CXCR4-S355D showed improved organization of ZO-1 (Fig. 6B) and VE-cadherin (Fig. 6C). However, CXCR4-S355A failed to recover endothelial junctional pattern due to the lack of phosphorylation at Ser355. Figure 6D recapitulated the effect of TBK1-CXCR4 on endothelial junctions.

### TBK1 was increased in human diabetic retinas

**Fig. 7:**
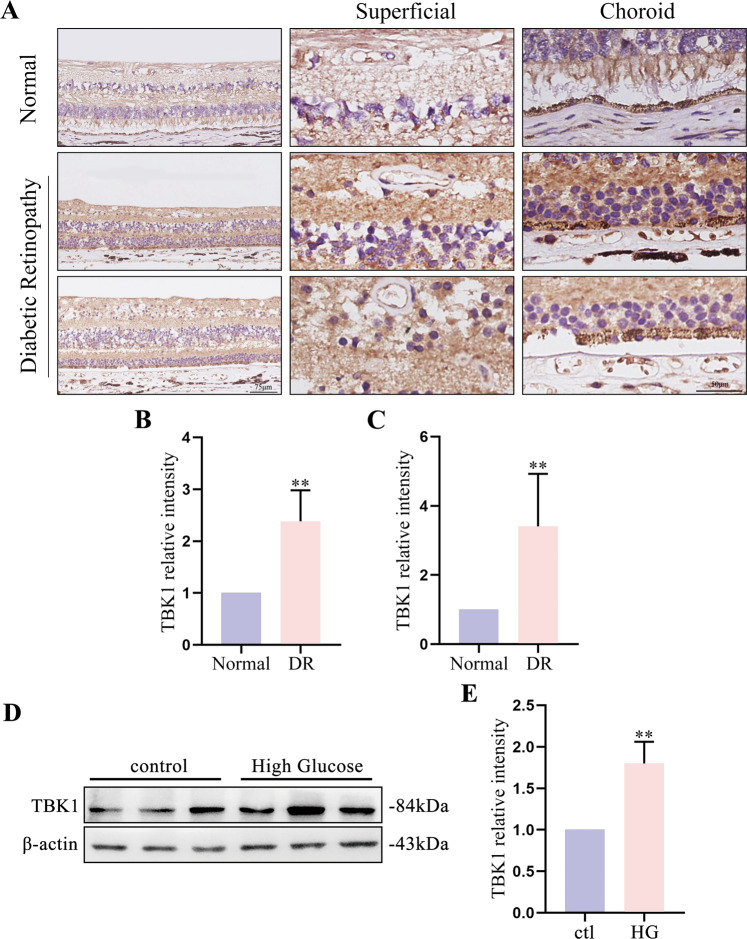
TBK1 expression was increased in human diabetic retinas. **A** Representative images of TBK1 immunostaining in human retinas. TBK1 was increased in retinal and choroidal vasculature with DR. **B** Statistical analysis of TBK1 expression in retinal superficial vessels in **A**. The data were means ± SD of three samples/group and assessed by student’s *t*-test. **C** Statistical analysis of TBK1 expression in choroidal vessels in **A**. The data were means ± SD of three samples/group and assessed by Welch’s *t*-test. **D** TBK1 expression in HRCECs was tested by western blot assay. Cells were treated with 30 mM glucose to establish a high glucose model. **E** Statistical analysis of the data shown in **D**. The data were means ± SD of three replicates/groups and assessed by student’s *t*-test. **P < 0.01.

DR is characterized by endothelial barrier dysfunction. Previous results revealed that TBK1 expression affected cell-to-cell junctions in endothelial cells. Here, we evaluated TBK1 expression in the retina samples of nondiabetic and diabetic donors. Increased TBK1 levels were observed in retinal superficial vessels and choroidal vasculature from diabetic donors (Fig. 7A–C). In addition, we treated HRCECs with high glucose, and found an increase in TBK1 expression (Fig. 7D, E). These results indicated the involvement of elevated TBK1 expression in DR.

## Discussion

The disruption of endothelial cell-to-cell junctions is critically involved in the pathogenesis of vasculopathy in ocular diseases. In this study, we have confirmed the role of TBK1 in endothelial cells, which is of great significance for retinal vascular development and barrier function. We provide powerful evidence that TBK1-mediated phosphorylation of CXCR4 at Ser355 plays a crucial role in cell-to-cell junctions. To our knowledge, this is the first report to comprehensively investigate the expression, function, and regulatory mechanism of TBK1 in endothelial cells by conditional knockout (CKO) mouse model combined with LC-MS/MS proteomic analysis.

It is known that complete deletion of *Tbk1* leads to embryonic lethality at day 14.5 due to liver degeneration and apoptosis [14, 15]. Therefore, further studies took advantage of the cre-loxp recombination system to generate *Tbk1* CKO mice models. Unexpectedly, they found that liver-specific *Tbk1* deficiency increased hepatic lipid accumulation without affecting inflammatory status [16]. By crossing the *Tbk1*^*F/F*^ mice with CD4-Cre mice, Yu et al. found that T-cell-specific *Tbk1* ablation mice showed splenomegaly as well as increased numbers of CD4^+^ and CD8^+^ T cells in lymphoid organs and the peripheral blood [17]. Other *Tbk1* CKO models were used to investigate the role of TBK1 in intestinal epithelial, dendritic cells, and myeloid cells [18–20]. However, the role of TBK1 in endothelial cells is still unclear. To evaluate the function of TBK1 in endothelial cells, we firstly generated endothelial *Tbk1* CKO mice (*Tbk1*^*ΔEC*^) by crossing the *Tbk1*^*F/F*^ with the *Tie2*-cre line. We found that endothelial *Tbk1* ablation compromised retinal vascular density, stability and permeability. These results suggest that TBK1 is involved in vascular development and barrier function.

TBK1 has crucial cellular functions, including the innate immune system, autophagy, proliferation, survival, insulin signaling, and metabolism. As an early effector of the innate immune system, TBK1 directly phosphorylates IRF3 and IRF7 on multiple serine and threonine residues, including Ser386, Ser396 on IRF3 and Ser477, Ser479 on IRF7 [21–24]. Kim et al. performed mass spectrometric analysis within TBK1 knockdown lung cancer cells, and reported 385 proteins with altered phosphorylation [25]. They found that TBK1 knockdown decreased metadherin phosphorylation at Ser568, which suggested metadherin as a downstream effector of TBK1 in lung cancer cell’s survival. In our study, we aimed to identify the novel and functional substrates of TBK1 in endothelial cells. LC-MS/MS proteomic analysis revealed 124 phosphorylation sites in 101 proteins with altered phosphorylation levels. We found and validated that endothelial TBK1 deprivation inhibited CXCR4 Ser355 phosphorylation.

Unexpectedly, we did not find the canonical substrate of TBK1 in our databases, such as IRF3, IRF7, or NF-κB signaling pathway relative proteins. One possible reason is that the proteomic analysis is based on the retinal samples of *Tbk1*^*F/F*^ and *Tbk1*^*ΔEC*^ mice. Indeed, the retinal tissue contains multiple cell types, including endothelial cells, glial cells, and neuronal cells. The alteration of phosphorylation level might be induced by endothelial TBK1 deletion directly or indirectly. In a further study, endothelial cells can be isolated and purified from *Tbk1*^*ΔEC*^ mice for proteomic analysis. The endothelial cells regulate vascular permeability through endothelial junctional proteins. The vascular endothelial growth factor (VEGF) induces phosphorylation of β-catenin and VE-cadherin, therefore decreasing junctional strength and increasing vascular permeability. In this study, we found that deletion of TBK1 disrupted endothelial junctions, which was reversed by phosphorylated CXCR4 Ser355 complement. We assumed that endothelial TBK1 manipulated vascular barrier integrity via CXCR4 phosphorylation regulation.

### Limitations of the study

CXCR4 plays a major role in multiple pathophysiological processes, such as inflammation [26], cell proliferation [27], and migration [28]. CXCR4 activates G protein-dependent signaling pathways, such as PI3K, AKT, EGFR, and mTOR [29, 30]. The signaling pathway of PI3K-AKT participates in the opening and sealing of endothelial tight junctions [31]. This study elucidated the interaction between TBK1 and CXCR4 in regulating the endothelial barrier, however, there are some possible limitations. First, it is unclear whether deletion of TBK1 disrupts endothelial junctions through CXCR4/PI3K/AKT pathway inhibition. Further study could investigate the relationship between CXCR4 Ser355 phosphorylation and PI3K/AKT pathway activation. Second, in this study, we only evaluated the role of TBK1 in endothelial junctions. Whether TBK1 deletion affects endothelial inflammation is indistinct. Third, the effect of TBK1 activation on vascular endothelial integrity remains to be uncovered in the future. It is possible that pharmacological activation of TBK1 improves CXCR4 phosphorylation at Ser355 and maintains endothelial permeability.

Taken together, we demonstrated that deletion of endothelial TBK1 caused retinal vascular leakage through CXCR4 Ser355 phosphorylation inhibition. This offers novel clues for the pathogenesis of BRB breakdown and provides new therapeutic targets for retinal vascular diseases.

## Methods

### Animals

*Tbk1*^*F/F*^ and *Tie2-Cre* mice were obtained from Gempharmatech (Nanjing, Jiangsu, China). Both lines were used to generate *Tbk1*^*ΔEC*^ and control littermates. Genotyping primers are as follows: the first *Tbk1*-loxP site, 5′-CAGATAGTCCATGTGGGTTCCG-3′ and 5′-TGACCCAGGGTCTCTTCACAAG-3′; the second *Tbk1*-loxP site, 5′-GCGTTACAGCCTAAGGAATGAGC-3′ and 5′-GCACAGCAGAGGCTTCTGAATG-3′.

All of the animal procedures were in accordance with the Association for Research in Vision and Ophthalmology (ARVO) Statement for the Use of Animals in Ophthalmic and Vision Research. They were also approved by the Use Committee of Huazhong University of Science and Technology.

### Cell culture and transfection

bEnd.3 cells (mouse brain microvascular endothelial cells) and HRCECs (human retinal capillary endothelial cells) were cultured in the complete endothelial cell medium (Procell, Wuhan, China). HEK293T were cultured in DMEM (Gibco, Gaithersburg, MD, USA) supplemented with 10% fetal bovine serum (Gibco) and 1% penicillin-streptomycin (Gibco) in a humidified atmosphere of 5% CO_2_ at 37 °C. Endothelial cells were infected with lentivirus carrying shTBK1 if necessary. Additionally, bEnd.3 cells were transfected with CXCR4-WT, CXCR4-S355A, or CXCR4-S355D plasmids with the assistance of Lipofectamine LTX & PLUS (Invitrogen). HEK293T were transfected with CXCR4-WT, CXCR4-S355A, or CXCR4-S355D plasmids using Lipofectamine 3000 (Invitrogen). Then cells were harvested 24 or 48 h after transfection as indicated for further analysis.

### Immunofluorescence

Preparing for cryosection, eyes were fixed with 4% paraformaldehyde (PFA) at room temperature for 4 h. Specimens were embedded in OCT compound (Sakura, Tokyo, Japan) and sectioned at 8 μm. For retinal whole-mount, eyes were fixed with 4% PFA at room temperature for 1 h. After dissection, retinas were transferred to cold methanol for further use. Preparing for cellular staining, endothelial cells seeded on glass coverslips were fixed in 4% PFA at room temperature for 15 min. Following fixation, samples were permeabilized with PBS containing 0.2% Triton X-100 for 30 min, blocked with PBS containing 3% bovine serum albumin for 1 h, and incubated with primary antibodies at 4 °C for 12–48 h. Primary antibodies used for immunofluorescence analysis were as follows: anti-TBK1 (Abcam, ab40676), FITC-isolectin B4 (IB4) (MilliporeSigma, L2895), anti-CD31 (Santa Cruz Biotechnology, sc376764), anti-collagen type IV (ab6586), anti-TER119 (R&D SYSTEMS, MAB1125), anti-VE-Cadherin (R&D SYSTEMS, AF1002; MAB9381), and anti-ZO-1 (Invitrogen, 33-9100). After wash out with PBS, samples were incubated with secondary antibodies at room temperature for 2 h. Images were captured using an inverted confocal microscope (Olympus FV3000).

### Western blot

Total proteins were extracted by RIPA Buffer (Beyotime) supplemented with protease inhibitor cocktails and phosphatase inhibitors (Sigma-Aldrich). Lysates were incubated at 100 °C for 5 min and centrifuged at 12,000 rpm for 15 min. After SDS-PAGE, samples were transferred to polyvinylidene fluoride (PVDF) membranes (Millipore). Subsequently, membranes were blocked in 5% skim milk at room temperature for 1 h and incubated at 4 °C overnight with antibodies raised against TBK1 (Abcam, ab40676), CXCR4 (Santa Cruz Biotechnology, sc53534), phosphoserine (Abclonal, AP0932), β-actin (Cell Signaling Technology, 3700), GFP (Proteintech, 50430), or His (Proteintech, 66005). Afterward, the membranes were incubated with horseradish peroxidase-conjugated secondary antibodies at room temperature for 2 h. A chemiluminescence signal was detected using the WesternBright ECL (Advansta) with Image Lab software.

### Coimmunoprecipitation (Co-IP) assay

Total proteins were extracted by RIPA Buffer (Beyotime) supplemented with protease inhibitor cocktails and phosphatase inhibitors (Sigma-Aldrich). The appropriate primary antibodies were added to the supernatant of the lysate for rotation at 4 °C overnight. Then, resuspended protein A/G agarose was added and incubated at 4 °C for 4 h. After wash with RIPA buffer, the immunoprecipitants were boiled in SDS-PAGE loading buffer, followed by western blot assays. The secondary antibodies were as follows: anti-mouse IgG LCS(Abbkine, A25012) and anti-rabbit IgG LCS (Abbkine, A25022).

### Evans blue assay

To investigate the vascular permeability, 1% Evans Blue dye was injected through the tail vein. After 1 h or 1 day as indicated, eyes were isolated for histological examination.

### OCTA imaging

Mice were anesthetized and fixed on a platform. The retinas were scanned with an imaging device for rodents (isOCT, Optoprobe, UK). The system operated at a central wavelength of 1060 nm with an A-scan rate of 200,000 Hz. The scan area was 2.5 mm × 2.5 mm, centered at the optic nerve head. Each OCTA volume was 600 A-scans by 600 B-scans, each an average of ten B-scans. The OCTA images were derived from the built-in software (RestrUI, Optoprobe, UK).

### Immunohistochemistry

Human retina samples were collected in the Tongji Hospital. The present study was approved by the Research Ethics Committee of the Tongji Hospital, Huazhong University of Science and Technology, and individual permission was obtained using standard informed consent procedures. The investigation conformed to the principles outlined in the Declaration of Helsinki regarding the use of human tissues. Preparing for immunohistochemistry, samples were fixed with 4% PFA, embedded in paraffin, and sectioned at 4 μm. After treatment with 3% H_2_O_2_ to eliminate endogenic peroxidase, the sections were incubated with an anti-TBK1 antibody (Abcam, ab40676). Thereafter, the sections were stained with horseradish peroxidase-conjugated secondary antibodies for 1 h. Images were captured using an Olympus BX53 microscope.

### Retina tissue isolation and sample preparation for mass spectrometry

The mice were sacrificed and the retinas were harvested. Retinal tissue samples were immediately isolated on ice. The tissue protein was extracted for subsequent experiments by using a lysis buffer (8 M urea, 1% Triton-100, 65 mM DTT, 1% Protease Inhibitor Cocktail, and PR619). Peptides were purified using reversed-phase Sep-Pack C18 cartridges and eluted with 50% acetonitrile. 1% eluted peptides were taken for proteome analysis. The protein contents were validated by sodium dodecyl sulfate–polyacrylamide gel electrophoresis (SDS-PAGE) with Coomassie blue staining.

### Digestion, fractionation, and affinity enrichment

Each tissue protein sample was sonicated three times on ice with a high-intensity ultrasonic processor (Scientz), and tandem mass tag (TMT) labeling was performed as follows. The digested peptides were desalted by a Strata X C18 SPE column (Phenomenex) and vacuum dried. The sample was reconstituted in 0.5 M triethylammonium bicarbonate and then processed with a 6-plex TMT kit according to the manufacturer’s instructions. It was then isolated into fractions with high pH reverse-phase high-performance liquid chromatography (Agilent 300 Extend C18 column with 5 μm particles, an internal diameter of 4.6 mm, and a length of 250 mm). The peptides were first separated into 60 fractions with a gradient of 8 to 32% acetonitrile in ammonium bicarbonate (10 mM, pH 9) for 60 min. They were combined into six fractions. Finally, the peptide mixtures were incubated with an IMAC microsphere suspension with vibration to obtain the whole enriched phosphopeptides for liquid chromatography-mass spectrometry (LC-MS)/ MS analysis.

### LC-MS/MS analysis

A Thermo Scientific™ Q Exactive™ Plus mass spectrometer was used for the identification of phosphorylation sites. Intact peptides were measured at a resolution of 60,000 in the spectrometer’s Orbitrap. The spray voltage was set to 2.3 kV, and automatic gain control was utilized to prevent the ion trap from overfilling. For the MS scans, the mass/ charge ratio (m/z) ranged from 400 to 1200, with the fixed first mass set as 110 m/z. MaxQuant with the Andromeda search engine (v1.4.1.2) integrated was selected for database searching of the resulting MS/MS data. The mass error was set at 10 ppm for precursor ions and 0.02 Da for fragment ions. The false discovery rate thresholds for proteins, peptides, and modification-sites were specified as 0.01.

### Bioinformatic analysis

Functional annotations were further performed by BLAST these proteins against the GO (http://www.geneontology.org/), KEGG (http://www.genome.jp/kegg/pathway.html), COG (http://www.ncbi.nlm.nih.gov/COG/), and eggNOG (http://eggnogdb.embl.de/). Meanwhile, we used the software WoLF PSORT to predict protein subcellular localization. GO and KEGG analyses were employed to test the enrichment of the differentially expressed proteins against all identified proteins, presenting the top ten items as histograms. Information about the phosphoproteomic dataset was summarized and analyses of differentially expressed sites cluster were visualized by a heatmap using the “heatmap.2” function from the “gpolts” R-package (Version 3.4.3). All differentially expressed protein database accession or sequence were searched against the STRING database (Version 11.0) for protein–protein interaction. To establish which kinases might phosphorylate these phosphopeptides, we analyzed the phosphorylation sites with the bioinformatic software tool Scansite. According to the high confidence score >0.7, the differential kinase–substrates interaction network was shown in the R-package “networkD3”.

### Statistical analysis

Data were represented as means ± SD from at least three independent experiments. Statistical analysis was performed using GraphPad Prism 8 software. The significance of the difference was evaluated using Student’s *t*-test or one-way ANOVA with the Bonferroni test as indicated in figure legends. *P* values < 0.05 were considered significant.

## Supplementary information

Original Data File

## Data Availability

All data generated or analyzed during this study are available from the corresponding author upon reasonable request.
