# Supplementary figures and images for: Endothelial deletion of TBK1 contributes to BRB dysfunction via CXCR4 phosphorylation suppression
Source: Cell Death Discov. 2022 Oct 28;8:429. doi: 10.1038/s41420-022-01222-y (PMC9616849; doi:10.1038/s41420-022-01222-y)

The original western blots used in figure 5 and 7.

5A

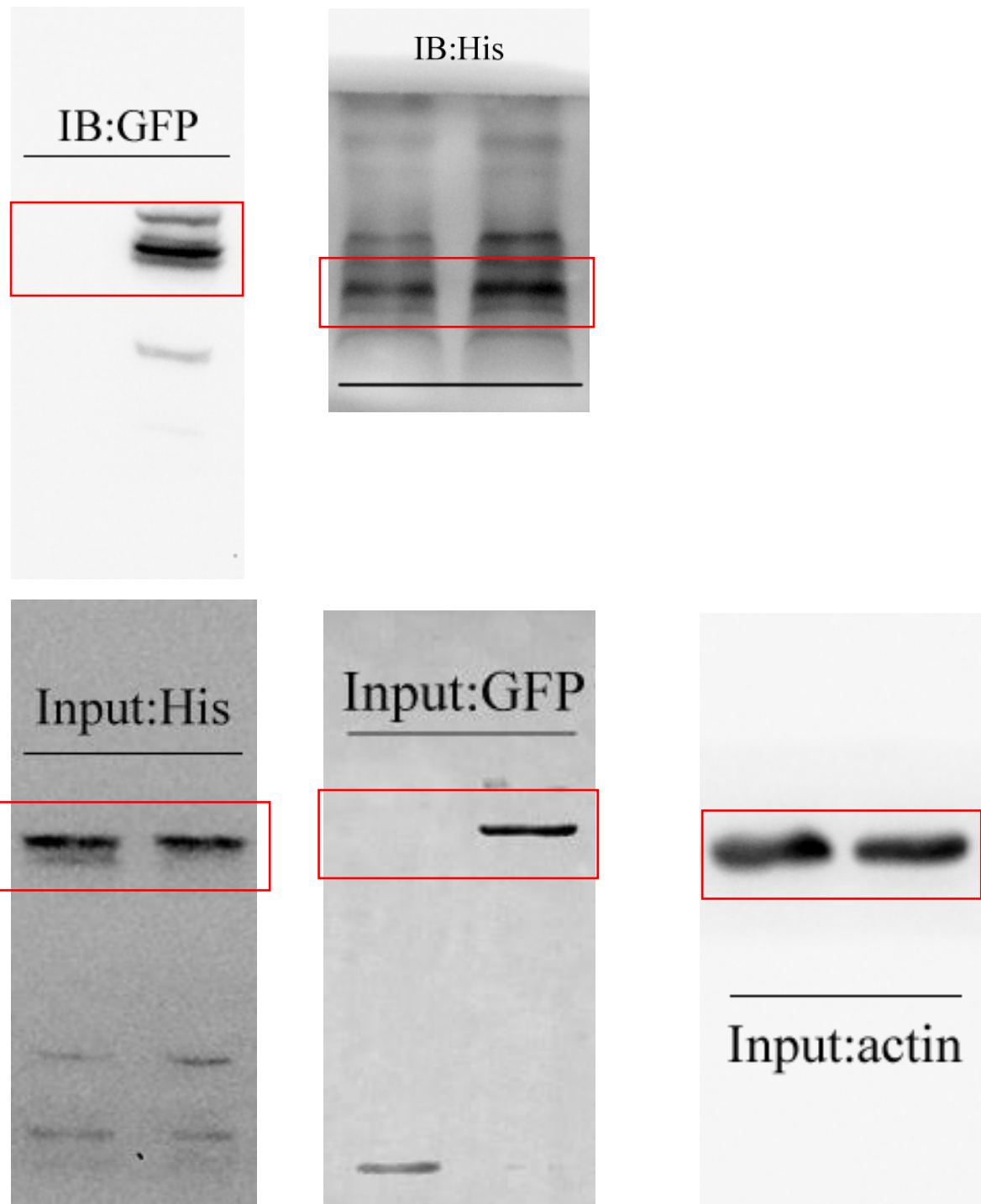

5B

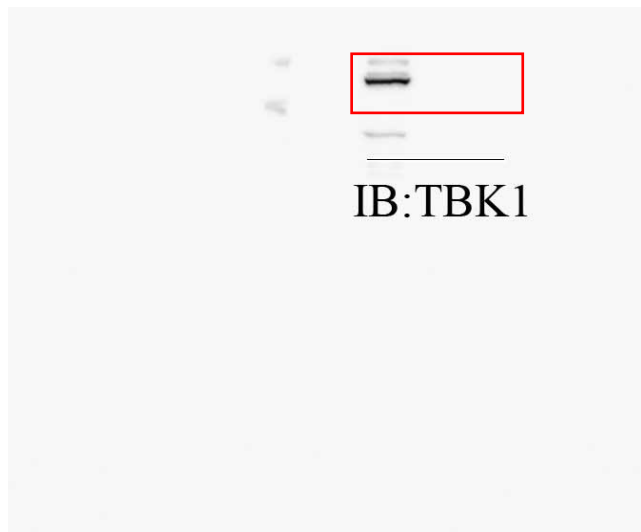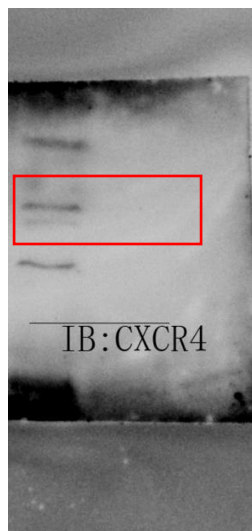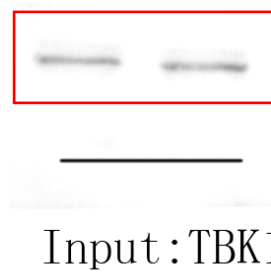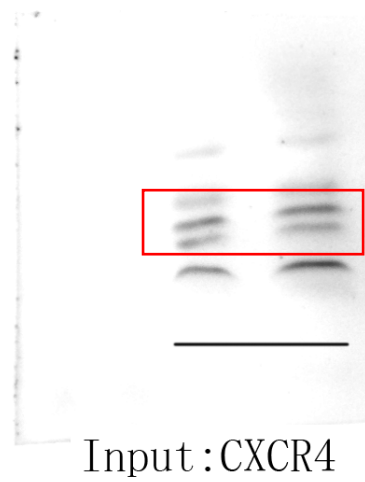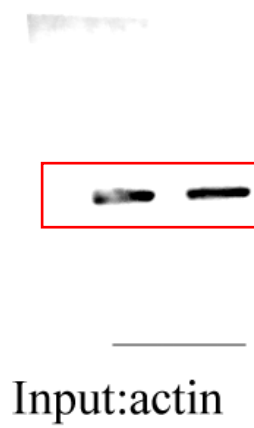

5C

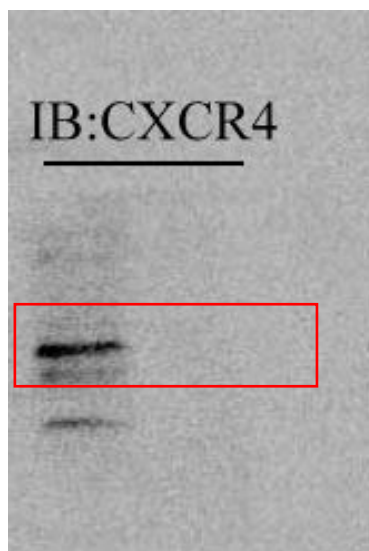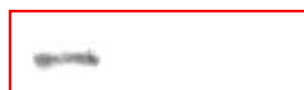

IB: TBK1

Input: TBK1

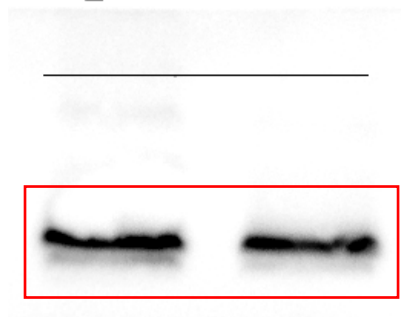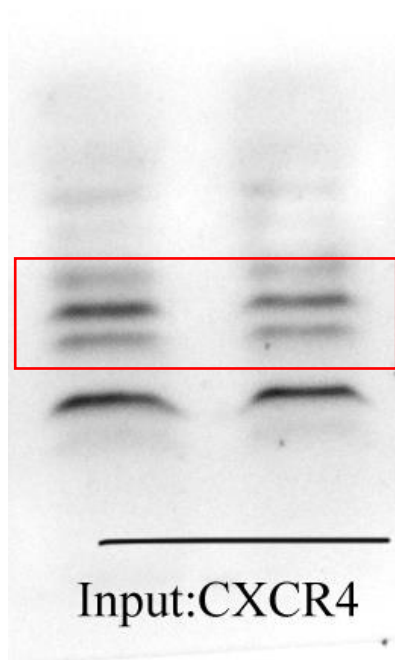

Input: CXCR4

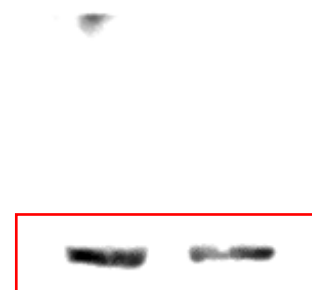

Input: actin

5D

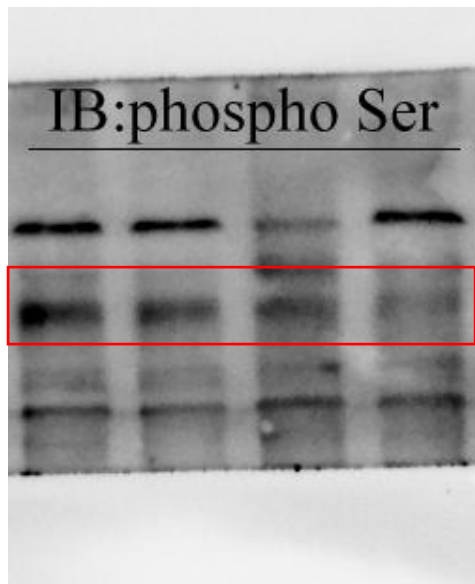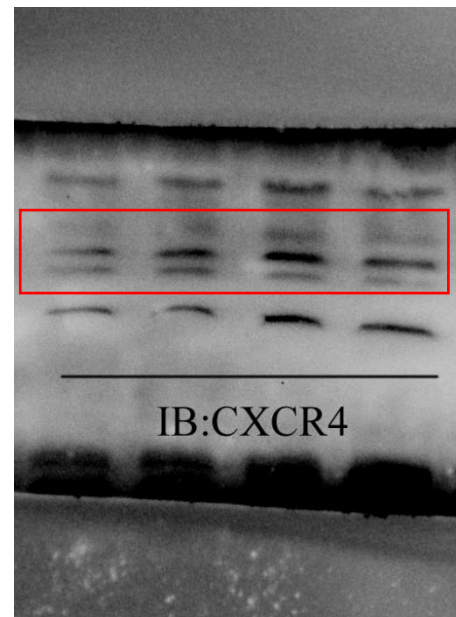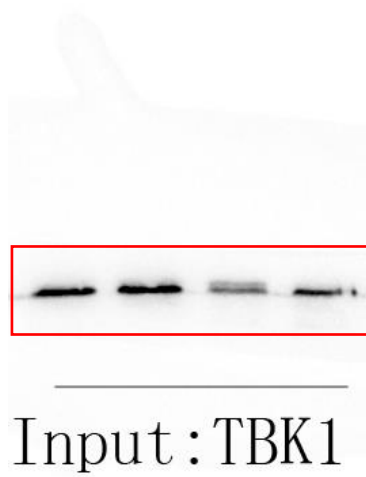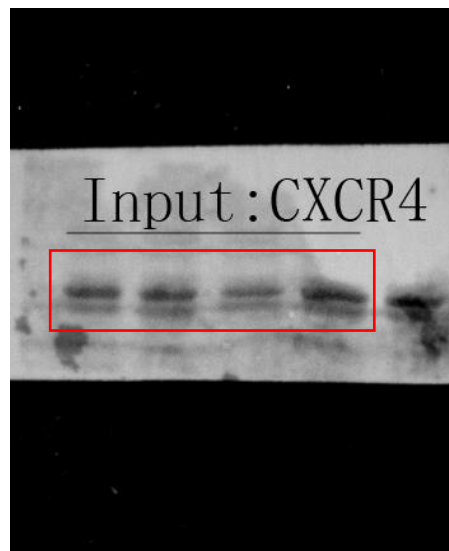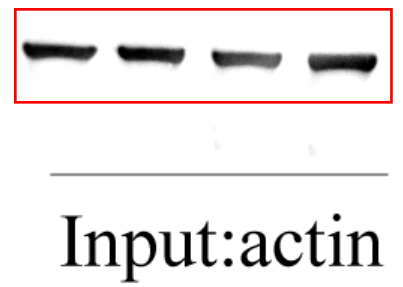

5E

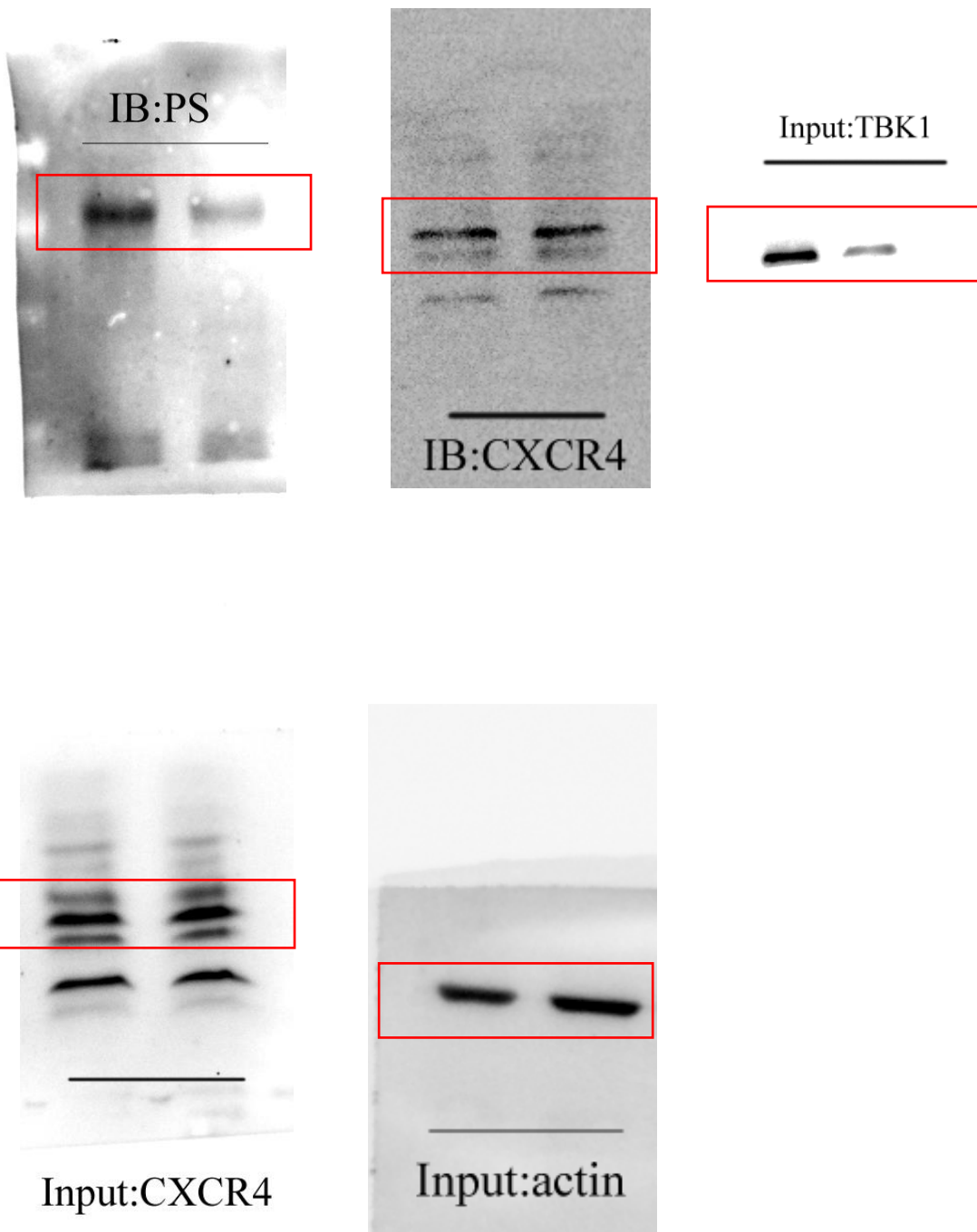

5F

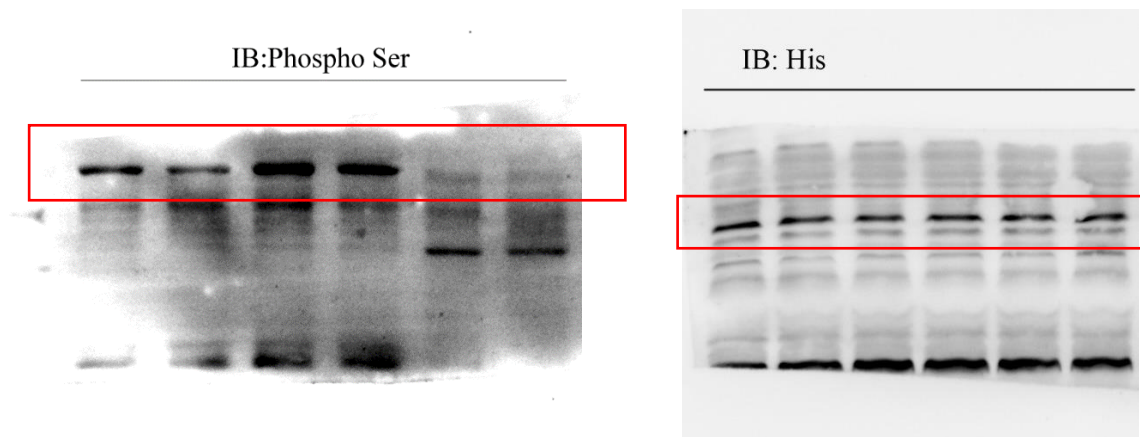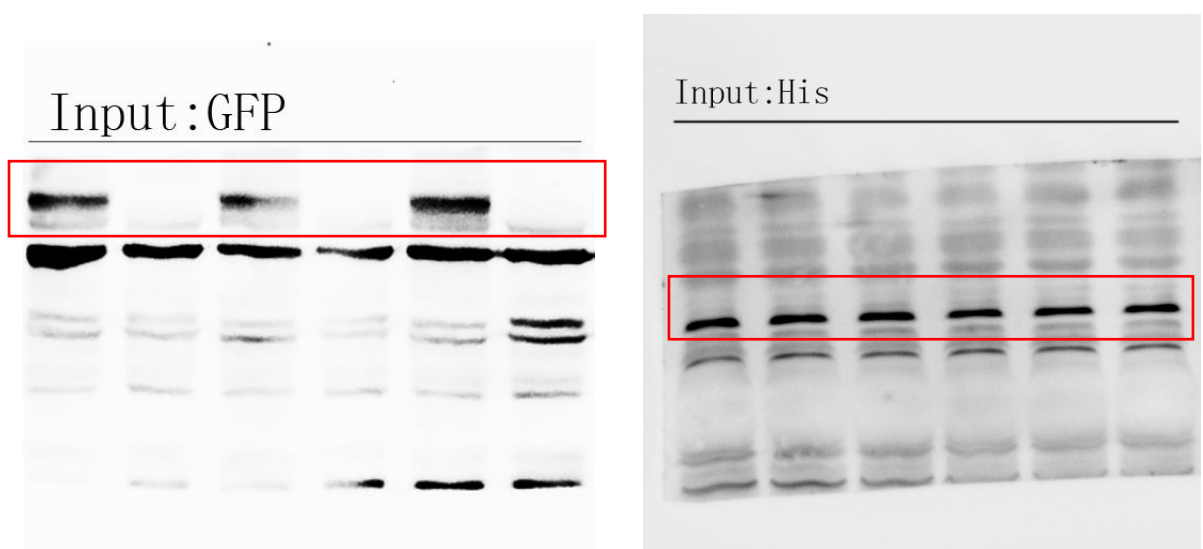

7D

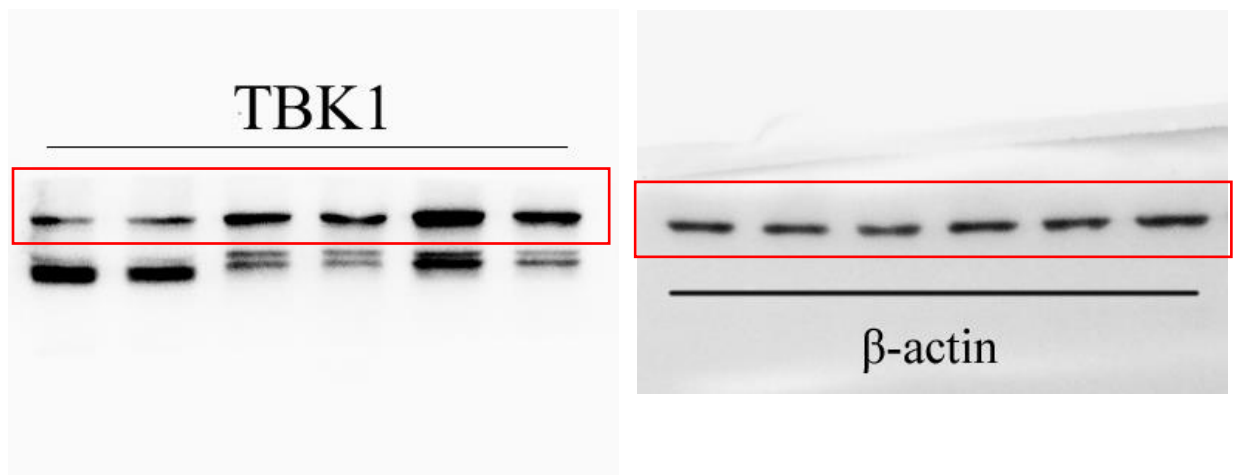

Supplement: Supplementary file 1 — Original Data File [file 41420_2022_1222_MOESM1_ESM.pdf]
